# Some Points of Preventive Treatment in the Diseases of Women

**Published:** 1899-06-17

**Authors:** Arthur E. Giles

**Affiliations:** Assistant Surgeon Chelsea Hospital for Women


### Hospital Clinics and Medical Progress.

### SOME POINTS OF PREVENTIVE TREATMENT IN THE DISEASES OF WOMEN.

By Arthur E. Giles, M.D., B.Sc., M.R.C.P.Lond., F.R.C.S.Ed., Assistant Surgeon Chelsea Hospital for Women.

(*Continued from page* 178.)

I come now to consider briefly a question of very great importance, Can cancer of the cervix be prevented? My belief is that it can in a large measure be prevented by the adoption of the principle I am now contending for, namely, by the early treatment of slight ailments. Let us see what facts are at our disposal in answering the question. Firstly, cancer of the cervix is much more common among married than among single women; secondly, it is more common in those who have borne children than in those who have not. The interpretation of these facts is that the injuries and diseases of the uterus associated with child-bearing predispose to the development of cancer. Further, in the case of the majority of women suffering from cancer, careful inquiry will elicit the information that there has been a previous history of inflammatory urine troubles. Now, the opinion is daily gaining ground that milder disorders of the uterus, if left untreated, may gradually change their character till they actually become the starting-point of the malignant growth. The deduction from these considerations is obvious and direct, namely, that by the early and thorough treatment of these milder disorders cancer might in some cases at least be prevented.

We may approach the question from another point of view. In the case of many conditions classed as "chronic endometritis" and "erosion," the microscopic appearance of the diseased tissues is not that of inflammation, but that of new growth, of the form known as adenoma or glandular hypertrophy. It is well known that the line of distinction between a benign and a malignant adenoma is often a very shadowy one; not infrequently neither the naked eye nor the microscopic appearance of a diseased cervix will allow us to say with certainty whether we have to deal with an erosion (adenoma) or with a commencing cancer. Consequently, it is not difficult to understand that an actual transition from the one condition to the other may readily occur. The deduction is as before, namely, that the milder disorders should be treated seriously, and nothing short of complete cure should satisfy us.

Although it is no part of my present purpose to enter exhaustively into details of treatment, I should like to put in a plea for the more rational treatment of "chronic endometritis" and "erosion of the cervix." As above stated, in many of these cases the pathological condition is not so much one of inflammation as of new growth; and by dealing with them as inflammatory, and treating them with caustics and cotton-wool, douches and tampons, much time is lost unnecessarily, much expense incurred by the patient, and much dissatisfaction caused to the practitioner. The secret of rapid and successful treatment is to recognise their true nature, and deal with them as new growths are dealt with elsewhere, viz., by removal. Thus adenomatous disease of the endometrium requires curetting; and "erosion," associated with laceration and ectropion of the cervix, demands removal of the diseased tissue and repair of the torn cervix by means of trachelorrhaphy.

**Conclusion.**—I have now said enough to indicate my purpose, and I shall have accomplished my object if by these remarks my medical brethren are induced to look into the matter afresh, and to direct their attention in some measure to the means of prevention; and if at the same time those outside our profession are led to recognise the extent to which preventive treatment lies in their hands. Advances in therapeutics and in surgery are daily giving us weapons wherewith to cope with disease that exists; but these very advances are achieved through doubt and discussion, with much conflict of ideas as to the relative merits of this or that drug or surgical procedure. And so, while earnestly welcoming every success recorded, I cannot but think that there is room for work and effort directed to prevention. To cure a dangerous condition is often a brilliant achievement, but to prevent it must be regarded as a much greater one, though it be received with less blowing of trumpets and blazoning of names on the roll of fame. The brilliant results are given to a few; the greater and therefore better results may be the lot of the honourable "rank and file," of those working conscientiously and unostentatiously for the relief of suffering and the safe-guarding of life.

